# Analysing synthesis of evidence in a systematic review in health professions education: observations on struggling beyond Kirkpatrick

**DOI:** 10.1080/10872981.2020.1731278

**Published:** 2020-03-31

**Authors:** Gillian Maudsley, David Taylor

**Affiliations:** a Department of Public Health & Policy, The University of Liverpool, Liverpool, UK; b Medical Education & Physiology, College of Medicine, Gulf Medical University, Ajman, United Arab Emirates

**Keywords:** Best evidence, cluster analysis, epistemology, evidence-based education, evidence synthesis, Kirkpatrick levels, Maxwell dimensions of quality, medical education, process analysis, systematic review

## Abstract

**Background**: Systematic reviews in health professions education may well under-report struggles to synthesize disparate evidence that defies standard quantitative approaches. This paper reports further process analysis in a previously reported systematic review about mobile devices on clinical placements.

**Objective**: For a troublesome systematic review: (1) Analyse further the distribution and reliability of classifying the evidence to Maxwell quality dimensions (beyond *‘Does it work?’*) and their overlap with Kirkpatrick K-levels. (2) Analyse how the abstracts represented those dimensions of the evidence-base. (3) Reflect on difficulties in synthesis and merits of Maxwell dimensions.

**Design**: Following integrative synthesis of 45 K2–K4 primary studies (by combined content–thematic analysis in the pragmatism paradigm): (1) Hierarchical cluster analysis explored overlap between Maxwell dimensions and K-levels. Independent and consensus-coding to Maxwell dimensions compared (using: percentages; kappa; McNemar hypothesis-testing) pre- vs post-discussion and (2) article abstract vs main body. (3) Narrative summary captured process difficulties and merits.

**Results**: (1) The largest cluster (five-cluster dendrogram) was acceptability–accessibility–K1–appropriateness–K3, with K1 and K4 widely separated. For article main bodies, independent coding agreed most for appropriateness (good; adjusted kappa = 0.78). Evidence increased significantly pre–post-discussion about acceptability (p = 0.008; 31/45→39/45), accessibility, and equity-ethics-professionalism. (2) Abstracts suggested efficiency significantly less than main bodies evidenced: 31.1% vs 44.4%, p = 0.031. 3) Challenges and merits emerged for before, during, and after the review.

**Conclusions**: There should be more systematic reporting of process analysis about difficulties synthesizing suboptimal evidence-bases. In this example, Maxwell dimensions were a useful framework beyond K-levels for classifying and synthesizing the evidence-base.

## Introduction

Synthesizing messy evidence in health professions education can be more art than science, more pragmatism than finesse, and more trouble apparently than it's worth. Nevertheless, exploring the evidence enough to make useful recommendations and to critique and improve the process must be better than investigatory inanition waiting for the perfect evidence-base.

Whether viewed as what is probably, plausibly, or often generally true, the nature of the ‘evidence’ [1] and the nature of its ‘good quality’ are debatable concepts. Approaches to synthesis are eclectic in Best Evidence Medical Education (BEME) systematic reviews [2]. In-depth consideration is limited though [3] about how to classify and analyse diverse evidence that thwarts standard quantitative approaches to the systematic review, and qualitative research synthesis is contested [4,5]. In making sense of a difficult synthesis, a post hoc standard veneer of slick reporting might hide how researchers cycle through confusion and clarity. While Kirkpatrick’s four-level outcomes-based model or taxonomy has been popular for imposing some order in systematic reviews in health professions education, it is useful to analyse how other frameworks might enhance the processes of organizing and synthesis.

### The popularity of the Kirkpatrick model

The Kirkpatrick model has been popular for straightforward, practical evaluation of training interventions by *K1 = reaction, K2 = learning, K3 = behaviour, and K4 = results* [6], especially in medical education [7,8]. Many BEME systematic reviews have used the model to filter and summarize [1], and its use to organize and synthesize systematic reviews and ‘quality-score’ quantitative evidence extends across diverse health-care education [9–11]. The common inferences that these ‘levels’ are a causal sequence (K1→K2→K3→K4) or hierarchical in value (K4>K3>K2>K1) have attracted criticism though [7,8].

Whether or not Kirkpatrick meant a causal hierarchy, he did view K1→K4 as increasing in complexity and meaning – thorough evaluation might use all levels [6]. Moreau [12] noted that the New World Kirkpatrick Model [13] improved on three criticisms: – *Difficulties evaluating K3/K4*: Evaluation now also focused on ways of promoting the application of learning (K3) and contributions to organizational goals (K4). – *Ignoring confounding and intervening variables*: Each K-level now included various personal and organizational influences. – *Inferring an unproven causal chain (K1*→*K2*→*K3*→*K4*): Considering ‘chains of evidence’ rather than implying a causal hierarchy of levels allowed non-sequential use of the levels. Such advances complemented re-thinking evidence synthesis beyond *‘effectiveness = Does it work?’*.

### Building beyond Kirkpatrick and ‘what works?’, using an example

The *‘What works?’* question could be dismissed as a narrow interpretation of effectiveness that uses only quantitative evidence for justification, or it could be interpreted more widely from whatever evidence supports *‘whether (it works)’* plus: *‘how, why, and in what circumstances (the context)?’. ‘The difficulty of evaluating any educational philosophy in a scientific manner’* occupied the early days of seeking best evidence (available) in medical (and other health professions) education [14, p.1]. The push to widen the horizons of BEME reviews has continued [15]. Systematic reviews in health services research and public health have long since been dealing with condensing complexity into concise counsel.

Petticrew [16, p.2] argued that the question for complex interventions should be:

“What has happened previously when this intervention [has] been implemented across a range of contexts, populations and subpopulations, and how have those effects come about?”

*‘Does it work?’* becomes *‘meaningless and usually unanswerable’* (p.2) for complex interventions, and narrative reviews seek evidence to reduce uncertainty rather than to derive a precise effect-size. Even weak studies provide illumination when a field is still in development and:

“evidence synthesis often is, and should be, an exercise in Bayesian decisionmaking, and reducing uncertainty, and not hypothesis testing” (p.5).

BEME review 52 [17] investigated: *‘What works best for health professions students using mobile (hand-held) devices for educational support on clinical placements?’* in an underdeveloped evidence-base. This was about a complex intervention and required versatile interpretation and classification of a mash-up of ‘whether’ (justification), ‘how/why’ (clarification), and ‘what’ (description) evidence [2,18]. Of the K2–K4 primary empirical studies included (K1-only studies were excluded), 46.7% (21/45) were mixed methods, 33.3% quantitative, and 20.0% qualitative research. K3 (86.7%) and S3-strength evidence (*Conclusions can probably be based on the results)* (55.6%) [19,20] predominated. There were only five L6 (randomized controlled trials) and two L5 (longitudinal) designs [21]. About three-quarters had supplementary K1 evidence and 53.3% had K4 evidence, mostly K4b. Inter-observer agreement on filtering abstracts was good (e.g. 92.1% in the final 2016 update, kappa = 0.64, p < 0.0001).

BEME review 52 concluded about mobile devices as educational support that [17]:

- They supported students’ learning on clinical placement via: assessment; communication; clinical decision-making; logbook or notetaking; and most often accessing information.
- In the hidden and informal curricula, ‘what happened’ was that students were:

- *bothered about*: actual and perceived disapproval of peers, clinicians or educators, and patients; confidentiality and privacy; and security aspects,
- *side-tracked by*: social connectivity (or other private use) and hectic clinical settings,
- *confused by*: policy ambiguity.

That review moved beyond *‘Does it work?’* and beyond K-levels. Much of the synthesis of the required *‘What works best …?’* question did implicitly answer *‘What has happened previously with use of mobile devices on clinical placements?’*. Maxwell’s dimensions from health services research [22,23] then helped to widen horizons. Used in evaluating quality of care, this *3As & 3Es* framework considers:

*Acceptability* (What do users prefer and how satisfied are they?). *Accessibility* (How reachable is the service? What are the barriers?). *Appropriateness* (How relevant is the service to needs?). *Effectiveness* (Does it work? What are the outcomes?). *Efficiency* (How are outputs to inputs? What are costs?). *Equity* (How fair is it?).

Adapting these dimensions (Table 1) helped to organize evidence and deliberate about synthesis. Previous BEME reviews did not feature this framework, warranting further analysis.

**Table 1.** Distribution and reliability: Maxwell dimensions (and final clustering with Kirkpatrick K-levels noted).A systematic review (Maudsley et al. 2019): *What works best for health professions students using mobile (hand-held) devices for educational support on clinical placements?* Evidence from n = 45 studies

In a preliminary analysis, BEME review 52 reported that the commonest Maxwell evidence-profiles (just under one-half) supported accessibility, appropriateness, acceptability, and effectiveness, or those plus efficiency, with little about equity-ethics-professionalism. Further analysis would gainfully explore the usefulness of that additional framework beyond Kirkpatrick, how both frameworks overlapped, and insights about presenting that body of evidence. As Regehr [24, p.34] argued:

“We are bound to learn more from our own work and that of others if we systematically examine and document our struggles than if we loudly proclaim our successes.”

In extracting transferable messages about the process of a systematic review, the aims here were to (1) Analyse further the distribution and reliability of classifying the evidence to Maxwell quality dimensions (beyond *‘Does it work?’*) and their overlap with K-levels. (2) Analyse how the abstracts represented those dimensions of the evidence-base. (3) Reflect on difficulties in the synthesis and merits of Maxwell dimensions.

## Materials & methods

Supplementary to BEME systematic review 52, the co-authors of this paper analysed their independent and consensus classification of evidence from the 45 primary studies [17: 3,228 ‘initial hits’ on 1988–2016 bibliographic database search]. This involved much immersion and deliberation on alternative interpretations, within the pragmatism paradigm [25]. These articles presented K2, K3, or K4 +/–K1 evidence, as K1-only articles had been excluded. ‘Self-reported’ evidence was allowed. For that review, a combined deductive content analysis and thematic analysis [3,26,27] focused on integrative synthesis to summarize the evidence systematically, quantifying as appropriate [28]. QSR NVivo 10 assisted data handling.

After calibrating 10 articles together, each reviewer also coded evidence in each article to one or more:

- K1-K4 and
- Maxwell dimensions of quality adapted to the mobile device (left column, Table 1): *acceptability, accessibility, appropriateness, effectiveness, efficiency, and equity* (expanded to equity-ethics-professionalism).

Disagreements were resolved by discussion. Discussion of these classifications also informed the overall integrative synthesis.

For each Maxwell dimension, analysis in IBM SPSS Statistics 24 calculated the percentage of the articles where, respectively, the main body presented or the abstract suggested empirical evidence. Cohen kappa measured the reliability of the independent Yes/No classifications of the main body (Reviewer 1 vs Reviewer 2). Imbalance in average prevalence of Yes vs No (near the extremes rather than 50%) [29,30, p.260] prompted use of prevalence-adjusted, bias-adjusted kappa (PABA-K) with 95% confidence interval.

Hypothesis-testing compared Yes-No for each Maxwell dimension from:

- combined pre-agreement (i.e. Yes = both independently coded to Yes; No = one or both coded to No) vs final agreed classification (post-discussion consensus) of main body of article.
- abstract vs main body.

McNemar hypothesis-testing treated both these comparisons as paired, under the null hypothesis of no difference in Yes-No.

**Figure 1.**
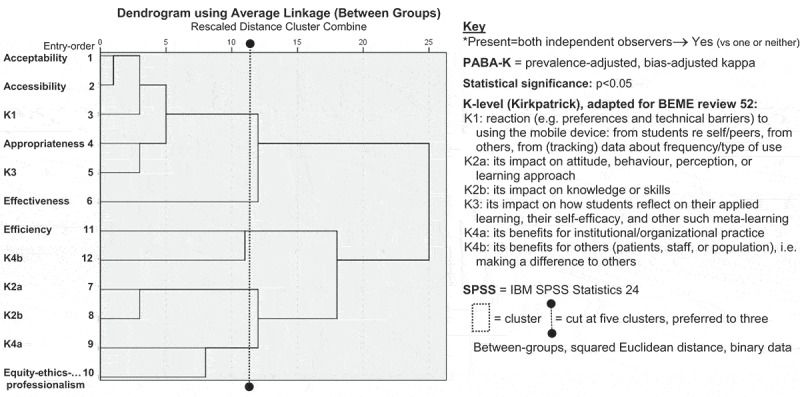
SPSS hierarchical five-cluster dendrogram: Maxwell dimensions and K-levels

SPSS 24 hierarchical cluster analysis explored how Maxwell dimensions and K-levels overlapped. An initial basic, simple-linkage, nearest-neighbour cluster analysis suggested the order for variables to enter a between-groups linkage analysis, clustering by variable (measuring Squared Euclidean distance; binary variable: 1 = Yes, 2 = No, rescaled to 0–1). A dendrogram summarized, from the left, stronger relationships with shorter horizontal fork-prongs, with vertical lines ‘joining’ variables (x-axis distance) (Figure 1) [31]. Coherence of findings and a ‘scree plot’ (agglomeration coefficient vs stage) suggested how many clusters to declare.

A systematic review [17]: *What works best for health professions students using mobile (hand-held) devices for educational support on clinical placements?* Evidence from n = 45 studies

Narrative summary captured reflection on the difficulties in synthesis [28]. Both reviewers independently outlined four main difficulties in synthesis and four main merits in using Maxwell dimensions in synthesis and analysis, then agreed a final summary in reflective discussion.

## Results

### Distribution and reliability of using Maxwell dimensions and overlap with K-levels

All bar one study provided evidence for appropriateness of mobile device use to learning needs (including caregiving and patient safety aspects) (44/45, 97.8%), 86.7% each for acceptability and accessibility, with 73.3% and 44.4% for effectiveness and efficiency, respectively, but only just over one-quarter for equity-ethics-professionalism (Table 1). For the main body of articles, independent observations of Maxwell dimensions agreed best for appropriateness (good, PABA-K = 0.78). Classifying to acceptability, accessibility, effectiveness, and equity-ethics-professionalism (mostly about digital professionalism) showed moderate agreement (PABA-K = 0.47–0.51). Classifying to efficiency reached only fair agreement (PABA-K = 0.33), broadly interpreted as consideration of outputs to inputs, such as costs, saving or making the most of time or effort in learning or providing care, or allowing timely feedback.

The largest cluster in the five-cluster dendrogram of K-levels and Maxwell dimensions was acceptability–accessibility–K1–appropriateness–K3, with effectiveness nearby, quite dissimilar from the efficiency–K4b cluster (Figure 1). K2a–K2b clustered stronger than K4a–equity-ethics-professionalism.

### How abstracts represented Maxwell quality dimensions of evidence

Acceptability was classified as ‘present’ significantly more post-discussion (same in 82.2%, p = 0.008; 31/45→39/45) and likewise for accessibility (same in 77.8%, p = 0.002; 29/45→39/45) and equity-ethics-professionalism (same in 75.6%; p = 0.001; 1/45→12/45).

If the abstract suggested evidence of a Maxwell dimension, the main body included that evidence. The main body presented evidence for acceptability and efficiency significantly more than the abstract: 86.7% vs 66.7%, p = 0.004; 44.4% vs 31.1%, p = 0.031, respectively. For accessibility, appropriateness, effectiveness (p = 0.063, respectively) and equity-ethics-professionalism (p = 0.250), the excess was not statistically significant.

### Reflecting on difficulties in synthesis and analysis and merits of Maxwell dimensions

**Figure 2.**
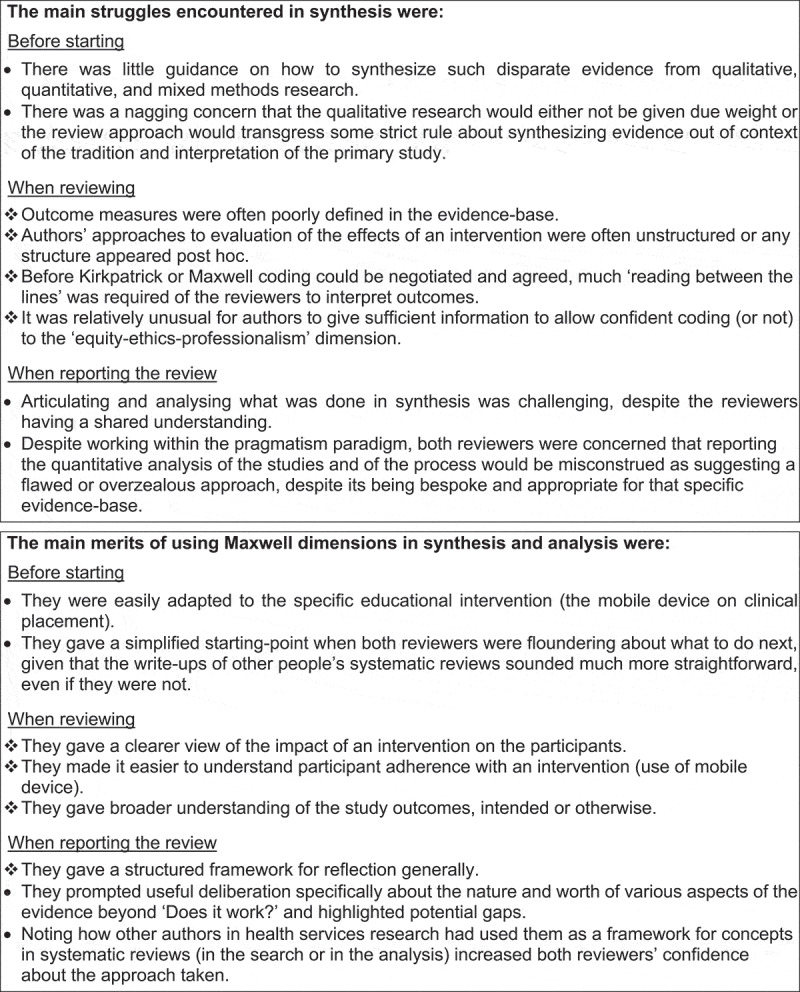
Two reviewers’ reflections (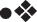) on struggles in synthesis and merits of Maxwell dimensions in synthesis and analysis

Combined observations about the main struggles in synthesis and analysis aggregated around the time (Figure 2):

- before: *insufficient guidance about such mixed evidence and concerns about transgressing qualitative research ‘rules’,*
- during: *ill-defined outcomes and methods, requiring much translation, particularly for equity-ethics-professionalism evidence,*
- after: *much effort in reporting analysis of the process and much potential to be misconstrued*.

A systematic review [17]: *What works best for health professions students using mobile (hand-held) devices for educational support on clinical placements?* Evidence from n = 45 studies

Likewise, for the list of Maxwell dimensions, main merits aggregated around the time (Figure 2):

- before: *a simplified starting-point, adaptable to the intervention,*
- during: *a lens for better and wider understanding of implementation and impact, intended or otherwise,*
- after: *a structured framework for reflection, prompting deliberation to consensus about tricky evidence and its wider worth (and possible gaps), and being consistent with application of Maxwell dimensions by systematic reviews elsewhere in health services research*.

## Discussion

Systematic reviews in medical education require more open discussion of difficulties in synthesizing suboptimal evidence-bases and more systematic reporting of process analysis. In the example explored here, Maxwell dimensions [22,23] helped to classify and synthesize the variegated quantitative, qualitative, and mixed methods evidence-base of a systematic review [17] meaningfully and moderately reliably, when only one-third of articles reported ‘quantitative-only’ research. The dimensions also helped to illuminate a coherent relationship with K-levels. Health professions education systematic reviews have not used this framework previously and very few health services research systematic reviews have reported using it [32–34]. As with the Kirkpatrick model, Maxwell dimensions were valuable in simplifying the approach [35] plus giving a deliberative framework for reviewers to share understanding in a tricky integrative synthesis and consider possible gaps. While the Maxwell list of ‘characteristics’ might not amount to a ‘framework’ for health-care performance measurement and improvement [36], here Maxwell dimensions provided a ‘framework for concepts’ of educational support. This built beyond *‘Does it work?’*.

When BEME review 52 [17] reported much potential for mobile devices to support health professions students on clinical placement (via their transitions, meta-learning, and care contribution, but requiring policy to address negative informal and hidden curricula), this explored beyond *‘Does it work?’*. Besides the usual recommendation for improved reporting of primary research, BEME review 52 recommended that ‘effectiveness’-reviews extend beyond a simplistic approach of just *‘What works?’*, echoing Eva [37] and Regehr [24]. This justified further scrutiny. This also reflected the broader horizons of health services evaluation [38] and challenges in systematic reviews of complex interventions in public health and social sciences [39,40], including the need to synthesize haphazard evidence. The ‘stainless steel’ law of such systematic reviews remained that *‘the more rigorous the review, the less evidence there will be to suggest that the intervention is effective’* [39, p.758]. In qualitative synthesis:

“study ﬁndings are systematically interpreted through a series of expert judgements to represent the meaning of the collected work. … the ﬁndings of qualitative studies – and sometimes mixed-methods and quantitative research – are pooled.” [3, p.253]

Better narrative synthesis is required [39] plus better blending with quantitative observations, as appropriate. Here, further analysis confirmed Maxwell dimensions to be a useful extra framework to prompt much deliberation on evidence, improve understanding, and represent complexity.

Maxwell dimensions typified health-care evaluation in the UK National Health Service (NHS) during its 1990s quality management and ‘internal’ market phase [41], providing a ‘characteristics model’ of quality [42]. In health services research, these dimensions have guided the integrative systematic review of *‘What is the effect of non-medical prescribing in primary care and community settings on patient outcomes?’*, exploring beyond *‘Does it work?’* [32]. A similar US Institute of Medicine [43] list of ‘characteristics’ (explicitly mentioning patient safety) was also popular, guiding classification of evidence in a systematic review of pay-for-performance in UK general practice [33]. While Donabedian’s much revered structure-process-outcome framework for measuring health-care quality might have been an alternative [44], it did not intuitively have as much potential for exploring the K-levels. Berwick and Fox [45] considered that the Donabedian framework was not necessarily patient-centred or viewing health care as a system. The Maxwell dimensions allowed the evidence in the educational context to be student-centred and to be viewed holistically as if part of a learning system. Several major NHS reforms and many alternative quality indicators later, more complex representations of quality have superseded Maxwell dimensions, e.g. to analyse patient-professional co-production of knowledge and health [46].

Nevertheless, Maxwell dimensions remain a basic, durable, practical starting-point for evaluating services [e.g. 47,48], notably dental in recent years [e.g. 49,50]. Relevant to workforce development, Halter et al.’s [34] systematic review of the impact of physician associates on secondary care used Maxwell dimensions as ‘outcome’ search-terms and then reportedly to organize the main messages (albeit without presenting or discussing the latter). Here, the dimensions adapted well to making sense of a research evidence mash-up about mobile devices in clinical placements for the future health professions workforce.

While the evidence is not obliged to represent all dimensions, the extra Maxwell lens highlighted potentially underrepresented aspects such as efficiency and equity-ethics-professionalism and an evidence distribution more towards the 3As. There was a gap in the evidence about quantifying efficiencies in learning or care provided. There was also a gap in the evidence about ‘fairness’ of the use of mobile devices.

Abstracts tended to omit much supplementary evidence about acceptability understandably (given exclusion of K1-only papers) but omitting evidence about efficiency suggested lower sensitivity of title-abstract filtering on this dimension. Despite moderately reliable independent observations, inter-observer discussion significantly increased the proportion classified to acceptability, accessibility, and especially equity-ethics-professionalism, probably reflecting refinement of definitions but also the subtlety of some evidence. A coherent clustering with K-levels confirmed that broadly interpreting ‘effectiveness’ across research types reached well beyond K2a-K2b randomized controlled trial–type evidence. Widening impact to ‘making a difference’ for the organization or for other people involved K4a and K4b clustering with, respectively, equity-ethics-professionalism and efficiency – and not with K1, so even if K-levels were non-hierarchical, this suggested that K1 and K4a/b differ substantively.

Yardley and Dornan [8] found ‘K2 and below’ to show suboptimal sensitivity as a BEME exclusion-filter for their review-question (about early workplace experience in undergraduate medical education). For BEME review 52 though, excluded K1-only papers did not illuminate its review-question further. Suitability of K-levels to filter and K-levels and Maxwell dimensions to organize and summarize trustworthy evidence depends on the review-question.

Strengths here were that both Kirkpatrick model and Maxwell dimensions were applied with critical scepticism and within the pragmatism paradigm, which enhanced: deliberation and synthesis; quantification of key aspects (Table 1, Figure 1); and mixing of qualitative and quantitative analytical approaches [51]. Furthermore, despite problematic calibration of Maxwell coding for equity-ethics-professionalism, the final coding appeared robust.

The evidence-base on which this ‘process analysis’ focused was relatively small, yet its eclecticism was both strength and weakness. Calibration about efficiency needed more attention. It was also unsurprising if evidence about acceptability and accessibility was uncommon, given exclusion of K1-only articles, but useful K1 supplementary evidence still featured. While using cluster analysis on 12 variables for *n* = 45 ignored a 2^m^ ‘rule-of-thumb’ sample-size (where m = number of variables) [31] and might be seen as overkill (or overreliant on hypothesis-testing [40]), the five-cluster dendrogram illuminated the Kirkpatrick–Maxwell relationship. Exploration of Maxwell coding against strength of evidence and study design may well also be merited but would require a larger evidence-base.

## Conclusion

Beyond the convenience of Kirkpatrick outcome-levels for filtering abstracts and summarizing outcome-evidence, Maxwell dimensions helped to promote Regehr’s [24] preferred imperatives for medical education evidence: *gaining a rich understanding* and *representing complexity*. This contrasted with what he called the dominant imperatives: *seeking proof*  [*‘that something works’*, 37, p. 295] and *generalizable simplicity*.

Reviewer-pairs or teams must calibrate and critique such classification tools with care to widen analytical horizons robustly. Here, deliberative synthesis of ‘whether’, ‘how/why’, and ‘what’ concepts [2,18,52,53] applied Maxwell dimensions to illuminate the process of implementing the educational intervention (using mobile devices on clinical placements) as well as broadly interpreting outcomes. To improve systematicity [54,55] and thoroughness for tricky integrative synthesis in systematic reviews, reflective deliberation and supplementary analyses about such tools are required, particularly their conceptual integrity and trustworthiness. Maxwell dimensions at least give a practical framework for organizing, deliberating about, and synthesizing key concepts when struggling beyond Kirkpatrick in a messy evidence-base. Waiting for the perfect evidence-base to synthesize would be unhelpful for the topic. As Glass [56, p.4] highlighted:

“A common method of integrating several studies with inconsistent findings is to carp on the design or analysis deficiencies of all but a few studies – those remaining frequently being one’s own work or that of one’s students or friends – and then advance the one or two ‘acceptable’ studies as the truth of the matter. This approach takes design and analysis too seriously, in my opinion. I don’t condone a poor job of either; but I also recognize that a study with a half dozen design and analysis flaws may still be valid. […] … I believe the difference [in results between poorly-designed and the best-designed studies] to be so small that to integrate research results by eliminating the ‘poorly done’ studies is to discard a vast amount of important data.”

Wilson and Lipsey [57, p.420] confirmed this in analysing 250 meta-analyses:

“It appears that low method quality functions more as error than as bias, reducing the confidence that can be placed in the findings but neither consistently over- nor underestimating program effects.”

In health professions education, it is challenging to undertake systematic multi-component mixed methods reviews that attempt to arrange, interpret, and summarize evidence about *‘What is the effect of this complex intervention?’* [58, p.2]. Such reviews may well attempt to ‘configure’ and (less so) ‘aggregate’ [58] an evidence-base that is quite a mess, epistemologically or otherwise. More tools and analysis of their use are required for the synthesis of jumbles of evidence.

## Practice points

- There should be more systematic reporting of process analysis about difficulties synthesizing suboptimal evidence-bases in health professions education.
- Maxwell dimensions are potentially useful for evaluating educational interventions and synthesizing messy, variegated evidence-bases.
- Kirkpatrick model and Maxwell dimensions applied to such an evidence-base clustered coherently together and should be applied with critical scepticism to improve understanding and represent complexity.
- To improve systematicity and thoroughness of systematic reviews, especially when synthesis is tricky, reflective deliberation and supplementary analyses about the conceptual integrity and trustworthiness of ‘filtering and classification’ tools are warranted.
- Be aware that abstracts may well omit certain types of substantive evidence reported in the main body, thus reducing the sensitivity of title-abstract filtering.
